# Development of a Hybrid Polymer-Based Microfluidic Platform for Culturing Hepatocytes towards Liver-on-a-Chip Applications

**DOI:** 10.3390/polym13193215

**Published:** 2021-09-23

**Authors:** Gulsim Kulsharova, Akbota Kurmangaliyeva, Elvira Darbayeva, Luis Rojas-Solórzano, Galiya Toxeitova

**Affiliations:** 1 School of Engineering and Digital Sciences, Nazarbayev University, Nur-Sultan 010000, Kazakhstan; elvira.darbayeva@nu.edu.kz (E.D.); luis.rojas@nu.edu.kz (L.R.-S.); galiya.toxeitova@nu.edu.kz (G.T.); 2 School of Sciences and Humanities, Nazarbayev University, Nur-Sultan 010000, Kazakhstan; akbota.kurmangaliyeva@alumni.nu.edu.kz

**Keywords:** microfluidics, microfluidic chip, liver-on-a-chip, microphysiological platform, hepatocytes, huh7, cyclic olefin copolymer, polydimethylsiloxane, hybrid polymer-based liver-on-a-chip

## Abstract

The drug development process can greatly benefit from liver-on-a-chip platforms aiming to recapitulate the physiology, mechanisms, and functionalities of liver cells in an in vitro environment. The liver is the most important organ in drug metabolism investigation. Here, we report the development of a hybrid cyclic olefin copolymer (COC) and polydimethylsiloxane (PDMS) microfluidic (HCP) platform to culture a Huh7 hepatoma cell line in dynamic conditions towards the development of a liver-on-a-chip system. The microfluidic platform is comprised of a COC bottom layer with a microchannel and PDMS-based flat top layer sandwiched together. The HCP device was applied for culturing Huh7 cells grown on a collagen-coated microchannel. A computational fluid dynamics modeling study was conducted for the HCP device design revealing the presence of air volume fraction in the chamber and methods for optimizing experimental handling of the device. The functionality and metabolic activity of perfusion culture were assessed by the secretion rates of albumin, urea, and cell viability visualization. The HCP device hepatic culture remained functional and intact for 24 h, as assessed by resulting levels of biomarkers similar to published studies on other in vitro and 2D cell models. The present results provide a proof-of-concept demonstration of the hybrid COC–PDMS microfluidic chip for successfully culturing a Huh7 hepatoma cell line, thus paving the path towards developing a liver-on-a-chip platform.

## 1. Introduction

Microphysiological platforms such as liver-on-a-chip (LOC) devices have the potential to reduce the costs of drug testing and the number of animal models used in drug development [1]. Perfusion in LOC platforms aims to emulate the in vivo dynamic flow environment in the liver. It can stimulate liver cell functions, including protein synthesis, carbohydrate transformations, detoxification, and hormone production in the human body [2]. Developing microfluidic chips that allow for successful cell growth and cell functionality under continuous flow is key in creating a robust LOC device.

Materials used to fabricate the platforms play a crucial role in their application for growing functional cell culture on-chip. Material considerations include biocompatibility with cells, optical transparency for imaging cell culture, suitability for rapid prototyping, and easy fabrication of microfluidic platforms [3]. One of the most commonly used materials in microfluidics and microphysiological platforms is polydimethylsiloxane (PDMS). It is low-cost, easy to use in soft lithography, optically transparent, highly elastic, gas permeable, and suitable for long-term cell culture in closed chambers [4]. However, its disadvantages, such as hydrophobicity and drug metabolites’ absorbance, have been well established [5]. As a result, there has been a growing shift in developing microphysiological platforms (MPS) towards other biocompatible polymers and alternative materials [4].

Among plastic polymers, cyclic olefin copolymer (COC) has excellent optical properties, low autofluorescence, and is FDA approved [3,6]. COC-based chips have been reported to show no adsorption compared to those made of PDMS [7]. Several COC-based microfluidic platforms for liver cell-based applications have been reported [6,8,9,10]. Rennet et al. reported a human liver model in a COC-based system with microfluidic flow conditions to study the influence of sepsis (severe inflammation) on hepatocellular dysfunction [9]. Wen et al. modeled a nonalcoholic fatty liver disease model with a cyclo-olefin polymer-based device seeded with a HepaRG hepatoma cell line [11]. Reported platforms have all been made solely of COC.

However, microfluidic devices made solely of COC could limit oxygen transport levels to cell culture inside the microchannels. While COC has higher gas permeability levels than other thermoplastics used for cell culture platforms (polymethylmethacrylate, polycarbonate) [7], it still cannot achieve the advantage in gas permeability levels of PDMS. This limitation complicates using COC-based microfluidic devices for most cell culture applications requiring high oxygen consumption [12]. For example, Bale et al. reported that within COC microfluidic microphysiological system, a decrease in oxygen concentration from 21% to ~10% was observed in contrast to another gas-permeable material [13]. Other studies with COC thermoplastic microfluidic devices have also shown a decrease in oxygen, resulting in hypoxia [14].

Hybrid microfluidic devices can be an alternative to combine the advantages of COC and PDMS while minimizing the described limitations of both materials. Hybrid microfluidic systems composed of different substrates are regarded as multifaceted and superior platforms [15]. Particularly, PDMS-based hybrid devices are able to avoid the limitations of those based solely on PDMS [15]. Combining COC, which has excellent properties, with PDMS results in fabricating reusable chips suitable for rapid prototyping while maintaining a gas-permeable environment in a cell culture chamber.

Hybrid COC and PDMS-based microfluidic devices have been reported for several applications, such as screening of lipidic mesophases [16], cell analysis [17,18], microfluidic mixing [19,20]. Among microphysiological systems, aorta-on-a-chip, a three-channel microfluidic system, where COC and PDMS were used as a middle layer in the system, was utilized to culture endothelial cells [21]. In particular, for liver-on-a-chip applications, a hybrid COC-based device in combination with another oxygen-permeable material (FEP) was reported for primary human hepatocyte (PHH) culture in recirculating flow conditions [22]. However, the used material, FEP, requires temperatures higher than 250 °C, so the material’s casting can be hazardous compared to using PDMS that can be cast at room temperatures [23]. While the device showed increased functions of the cultured PHHs in flow, the platform is specifically designed for high-throughput screening based on 96-well individual wells and pumps. Thus, the design requires a more complex fabrication which is not necessary for many applications, as in the case of a quick assessment of cell culture growth in a perfusion environment. Furthermore, the use of immortalized cell lines as a cell source in many LOC applications is preferred since using PHHs negatively impacts the reproducibility of the results due to their extraction and donor-to-donor variability [24]. Thus, hybrid platforms based on COC and PDMS that are easy to fabricate may be more preferable for initial proof-of-concept experiments, such as culturing immortalized cell lines in fluidic conditions.

Here, we present the development of a hybrid microfluidic chip that takes advantage of both COC and PDMS polymers. The COC layer of the HCP allows for optical transparency, biocompatibility essential for imaging, while the PDMS enables better gas permeability to perfusion culture. The hybrid COC–PDMS device (HCP), suitable for rapid prototyping and reusability, is applied for culturing functional hepatoma Huh7 cell line, paving the way towards the development of a liver-on-a-chip device. To the best of our knowledge, this is the first work utilizing COC–PDMS-based microfluidic design for a culture of an immortalized Huh7 hepatoma cell line in a perfusion environment. We present the biocompatibility evaluation of polymers used to build the HCP on the growth of Huh7 cells. We present results from computational fluid dynamics modeling to evaluate the air-volume fraction produced in the HCP microchannels and the flow distribution inside the device. The growth and cell functionality of Huh7 cells in the HCP perfusion system are carried out and analyzed through monitoring the secretion rates of albumin, urea, and final cell viability. The presented HCP platform can be used for any cell line for cell culture growth and for testing various conditions in a dynamic fluidic environment.

## 2. Materials and Methods

### 2.1. Materials

Cyclic olefin copolymer Topas microscopy slides were obtained from ChipShop, Germany (10-0678-0347-2.0-02). Polycarbonate sheets were obtained from Polycarbo, Russia. The human hepatoma Huh7 cell line (ATCC) was kindly provided by Dr. Mukhatayev, Nazarbayev University, Kazakhstan. Human Albumin ELISA kit (EHALB), Urea Nitrogen Colorimetric detection kit (EIABUN), collagen-coated flasks, and well plates were purchased from ThermoFisher Scientific (Waltham, MA, USA). All other materials and kits were purchased from Sigma-Aldrich (Hamburg, Germany) unless mentioned otherwise.

### 2.2. Microfluidic Chips Design and Fabrication

**Figure 1:**
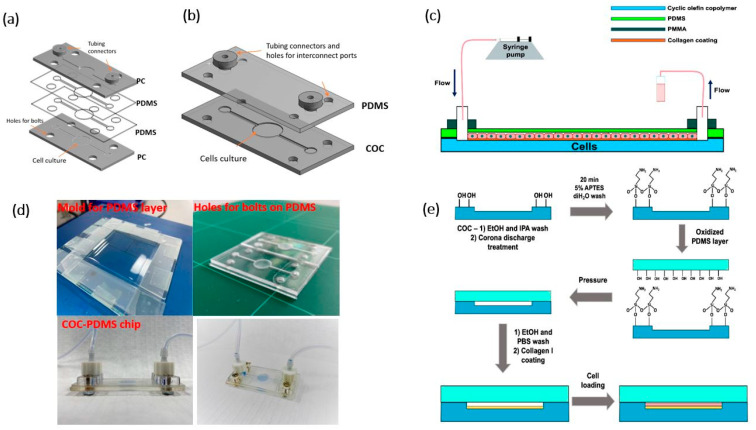
(**a**,**b**) Assembly view of a polycarbonate–polymethylsiloxane (PDMS) and a hybrid cyclic olefin copolymer (COC)–PDMS (HCP) microfluidic chips for culturing Huh7 hepatoma cell line; (**c**) Continuous flow experimental setup; (**d**) Fabrication of a PDMS layer and an image of the assembled HCP chip; (**e**) Schematic illustration of a COC–PDMS bonding and preparation of the microfluidic device seeded with cell culture.

Microfluidic chips (5.5 cm × 2.5 cm) and interconnect ports were designed using CAD software (Figure 1a,b). Chips were fabricated using polycarbonate (PC)–PDMS and COC–PDMS pairs using milling machine DMU-50 (DMG Mori, Winterthur, Switzerland) and casting PDMS. The outside and central chamber dimensions of the chips were adapted from an organ-on-a-chip platform (MesoBiotech, Paris, France).

A PC–PDMS chip shown in Figure 1a consisted of two PDMS (SYLGARD^®^184) layers sandwiched between upper and lower PC bases and bonded to the PC by corona surface treatment by laboratory corona treater (Electro-Technic Products, Chicago, IL, USA). All layers had the same microchannel pattern, either milled or cut out (depth of 500 μm and a width of 1 mm). Figure 1b shows a schematic of a hybrid COC–PDMS (HCP) microfluidic chip made of COC with a microchannel and a PDMS layer with a flat surface. A microchannel with a depth of 500 μm and a width of 1 mm was milled on the COC microscopy slide. The HCP chip (Figure 1d) was then assembled using the fabricated COC layer and a 1 mm PDMS layer.

A PDMS mold was made in-house using glass and microscopy slides to ensure no leakage (Figure 1d). The mold was filled with a calculated amount of polymeric base and a curing agent with the ratio 10:1, respectively, and held at 100 °C for 1.5 h. The resulting layer was then cut to the microfluidic channel dimensions in the case of the PC–PDMS chip, while for the HCP, holes for bolts, inlet, and outlet were punched through.

The bonding procedure for COC/PDMS was adapted from the literature [25]. Under aseptic conditions in a biological safety cabinet, the COC layer with the microchannel was treated with Corona discharge for 1.5 min and submerged in 5% APTES (3-aminopropyl triethoxysilane) for 20 min, then rinsed in sterile DI water. The PDMS layer was subjected to Corona discharge treatment for 1.5 min, and the COC chip base was bonded to the PDMS layer. Next, the channels and the well were washed with 70% ethanol and 1 × phosphate-buffered saline (PBS). The culture surface was coated with a 0.01% collagen Type I solution, sterilized under a UV lamp before loading to the chip with a pipette. The solution was allowed to bind to the surface for several hours and then was dried as per the manufacturer’s instructions (Sigma-Aldrich, Hamburg, Germany). The resultant surface coverage was about 5 μg/cm^2^ (calculations are provided in the Supplementary Materials section). The coated chip was washed with 1× PBS before loading the cells.

Interconnect ports were fabricated using a laser cutting machine CLD-1325 (Epilog, Golden, CO, USA) from a 4 mm thick PMMA. The resulting device was tapped with two M3-threaded connecting holes and one M4-threaded orifice to attach the platinum-cured silicone tubing (1 mm, Darwin Microfluidics).

### 2.3. Substrate Biocompatibility Studies

Polycarbonate (PC), cyclic olefin copolymer (COC), and polydimethylsiloxane (PDMS) substrates were assessed for biocompatibility. PC wells with a diameter of 34.6 mm were fabricated out of 5 mm polycarbonate sheets via milling. COC was used as open slabs and as the lower layer of the HCP chip in collagen-coated form. PDMS was used as a sole substrate and as the top layer of the HCP microfluidic chip with a thickness of 1 mm and 3 mm bonded to the COC base, separately. COC and PC microchannels were fabricated following the same procedure as described in Section 2.2. PC and COC substrates were coated with collagen as described in Section 2.2. Standard polystyrene plates in bare (for PC and PDMS) and collagen-coated forms (for COC) have been used for controls as a traditional platform. Huh7 cells were cultured on the substrates, and the cell attachment and viability were assessed utilizing optical and fluorescence microscopy imaging as well as cell count using a hemocytometer.

### 2.4. Microchannel Characterization

A fabricated microfluidic chip was connected to a syringe pump (AL 1000-220) to determine the reactor volume. Water was pumped at a 10 μL·min^−1^ flow rate, starting from an empty microchannel until the water reached the outlet. The reactor volume, then, was calculated by multiplying the used flow rate by the processing time.

The contact angle measurements were performed on an OCA 15EC optical contact angle measuring device (DataPhysics Instruments, Filderstadt, Germany). The profile of the channel and well surface was made using Dektak XT Stylus Profiler (Bruker, Billerica, MA, USA).

### 2.5. Cell Culture and Material Biocompatibility

Huh7 cells were maintained in a T-25 flask in 1× Dulbecco’s Modified Eagle Medium (DMEM), 10% FBS, 1% penicillin/streptomycin, 1% non-essential amino acids, in a 5% CO_2_ incubator at 37 °C for 24 h after thawing. After 24 h, cells were used for the flow experiments. The control batches were cultured on collagen-coated T25 flasks with the same cell seeding density as in the flow experiments.

### 2.6. Cell Loading and Flow Experiments

The fabricated COC–PDMS or HCP chip was used for flow experiments. The cells were loaded into a preliminarily collagen-coated chip and cultured in the chamber for 1 day before flow experiments at a density of 675 cells·mm^−2^. An Aladdin Single-syringe Pump (World Precision Instruments, Tregoland OU, Tallinn, Estonia) was used to supply the cells with cell culture media at a rate of 3 μL·min^−1^ (Figure 1c). The supernatant was collected in 300 μL volume fractions for 24 h every 100 min.

### 2.7. Cell Functionality Assessment

Cell morphology and metabolic activity were analyzed via microscopy and ELISA assays to determine the secretion rate of hepatic biomarkers in the chip in flow-through media.

The flow-through fractions obtained over 24 h of the experimental period were analyzed for albumin and urea secretion rates using ELISA according to the Invitrogen manual. All samples were analyzed in duplicates. For the urea detection, to account for the phenol red readouts in DMEM, the absorbance values of the DMEM control were subtracted from the data points. The plates were read at 450 nm on a Varioskan Flash microplate reader (Thermo Scientific, Waltham, MA, USA) for both assays.

Cell viability was confirmed at the end of the flow experiment after the samples were stained with a live/dead staining kit based on Calcein-AM and Propidium Iodide, according to the protocol provided by Sigma-Aldrich.

### 2.8. Microscopy

Optical and fluorescent images of the cell culture were obtained using the ZOE Fluorescent Cell Imager (Bio-Rad, Hercules, CA, USA).

## 3. Results and Discussions

### 3.1. Evaluation of Microfluidic Device Substrates

A short experimental testing study was carried out to verify that the substrates are suitable for cell growth. Overall, the biocompatibility and adsorption properties of PC, COC, and PDMS are well known and have previously been reported in the literature [3,7]. Based on previous reports, these materials were considered in this work due to biocompatibility, transparency, and their suitability for rapid prototyping. Initially, short testing experiments were conducted to confirm the cell growth on these polymer substrates, which were analyzed mainly through microscopy and PC through cell counting. In polycarbonate and COC, better cell adherence and growth were observed when the substrates were preliminarily coated with collagen than their bare counterparts. Collagen is a type of triple-helix structure protein that can initiate and maintain the interaction between cells and the matrix [26]. Collagen type I, the most common type of protein [26], has been used in this work. In PDMS, coating it with collagen was not considered, as the PDMS is used only as a top layer of microfluidic devices, and the design does not require cell placement on this material.

A lower layer of the PC–PDMS chip shown in Figure 1a was made of polycarbonate. However, Huh7 cells were initially grown on in-house milled PC wells milled out of 5 mm PC sheets and in the bottom PC part of the PC–PDMS chip separately. Bare polystyrene dishes were used for growing the control batch. Cell growth on a collagen-coated PC well was 117.8% on bare polystyrene control and 108.1% on a non-coated PC substrate (Figure S1). The standard doubling time of the Huh7 hepatoma cell line is generally reported as 24 h [27]. As mentioned, collagen facilitates the interaction between the cells and the matrix. The collagen-coated PC showed the best cell growth, and PC biocompatibility results were in line with those reported in the literature [3,7]. The results obtained allowed considering the design based on PC (Figure 1a) for testing in fluidic studies, but these studies showed disadvantages from a robustness point of view discussed later.

Biocompatibility and absorbance of PDMS and COC have been extensively investigated and reported earlier in the literature [4,7]. PDMS is the most widely used material for the majority of the reported organ-on-a-chip devices [28,29]. Cell growth on the PDMS layer was carried out to confirm that the material is not toxic to the cells.

PDMS may be limited from achieving high interaction with cells due to its natural hydrophobicity [30], and microfluidic structures made from PDMS can be subjected to dimensional distortions due to its low elastic modulus [31]. However, microscopy imaging of PDMS layers as a substrate for cell culture showed that PDMS was biocompatible with cells and was able to maintain cell growth (Figure S2). PDMS is known to be biocompatible, and cell viability results are reported in the literature [7]; therefore, quantitative cell viability studies were not carried out in this work, particularly because cells are not grown directly on the PDMS but rather on the bottom parts of the investigated chip designs.

**Figure 2:**
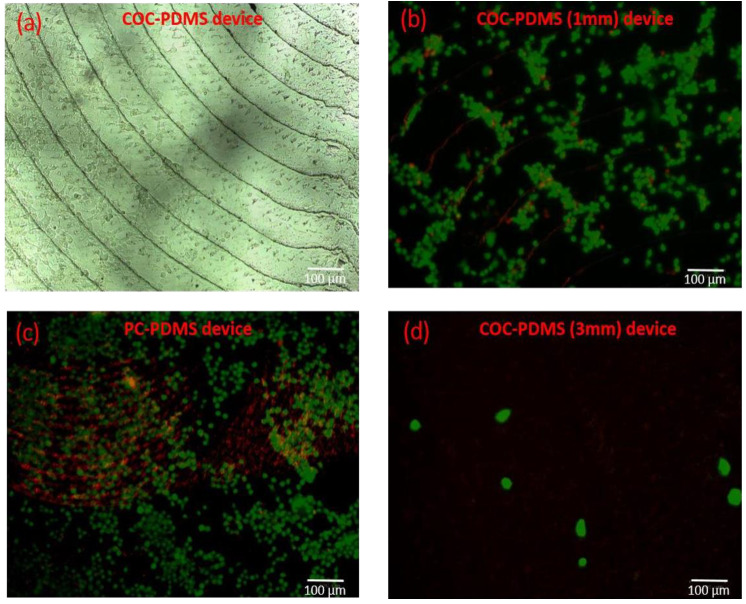
Microscopy images of the Huh7 hepatoma cell line cultured in the fabricated devices: (**a**) Optical image of Huh7 cells grown in the COC–PDMS device (Figure 1b) with a thickness of 1mm for 24 h. The image was taken by 10× objective; (**b**) Live-dead cell imaging results from the COC–PDMS device with 1 mm thickness of the PDMS layer. Green (Calcein-AM)—live cells, red (Propidium Iodide)—dead cells. (no autofluorescence from the substrate); (**c**) Live-dead cell assay images on the PC–PDMS device (Figure 1a) resulting in autofluorescence from PC; (**d**) Live-dead assay imaging of the COC–PDMS device with 3 mm thickness of the PDMS layer. Experimental results showed a possible lack of oxygen through a thick layer of PDMS, resulting in almost zero cell viability. At low cell densities, cells tend to change morphology; therefore, cells seem visibly bigger in size on this image.

Cyclic olefin copolymer (COC) was considered a potential material for microfluidic devices in this work due to its applicability for rapid prototyping similar to PC, PDMS, and its high melting temperature suitable for autoclaving for cell culture applications, and many other advantages [4]. Since COC is used as the bottom part of the COC–PDMS (Figure 1b) chip, COC substrates were coated with collagen due to the favorable advantages of collagen to cell culture [26]. Microscopy imaging of cell growth on collagen-coated COC slabs (Figure S3) and the collagen-coated chip microchannel in the lower part of the HCP chip (Figure 2a) confirmed the biocompatible nature of the COC substrate as expected and as previously reported in the literature [7]. From the microscopy images, cell growth on COC resulted as expected and was comparable to standard control substrates used for tissue cultures (Figure S3b). COC shows beneficial properties over other thermoplastics [32]. Therefore, from a biocompatibility point of view and due to many other attributes of COC, it was chosen over the PC to be used as the bottom part of a microfluidic chip in the HCP design.

Optical images of Huh7 cells cultured in the HCP device (Figure 2a) and live-dead cell assay imaging allowed investigating cell functionality in the PC–PDMS and COC–PDMS microfluidic devices. In the PC–PDMS device, autofluorescence due to PC was observed (Figure 2c), which inhibited microscopy imaging of dead cells in this chip. The autofluorescence observed when using PC as a substrate was expected since it was reported in previous studies on tissue culturing [7]. Additionally, the PC–PDMS chip showed limitation in reuse as leakage under low but long continuous flow of 5 μL·min^−1^ often resulted from the boundaries. In contrast, the fabricated COC–PDMS device showed minimum autofluorescence (Figure 2b) and was more suitable for further fluidic experiments.

### 3.2. Hybrid COC–PDMS Device Overview

The fabricated hybrid COC and PDMS (HCP) chip allowed portability, optical transparency, and reusability. The concept of the HCP design is based on cell attachment on the COC layer coated with collagen, while the PDMS layer provides additional advantages of possible gas permeability to the cell culture. The COC–PDMS chip was suitable for rapid prototyping. The microchannel depth was measured using a contact profilometer (Figure S4) and was found to be 450 μm, which was in the range of the initially developed design (500 μm). The calculated reactor volume was 131 μL, and the experimental measure was (137 ± 5) μL, determined as described in Section 2.4. At the same time, the irreversible sealing between the layers allowed a stable and robust system, which prevented any possible leakage through the two layers during the cell culture experiments.

It has been shown that continuous perfusion has positive influence on long-term cell survival [33]. However, at the same time, high shear stress caused by high flow rates may negatively impact cell morphology and adhesion to the surface. When we tested the performance of the HCP chip under high flow rates, cell detachment when imaged after 5–6 h of flow was observed. Additionally, at flow rates higher than 10 μL·min^−1^, bubble formation in the circular wells was observed. At low flow rates of 3–5 μL·min^−1^, no visible air bubbles were formed. Therefore, we utilized computational fluid dynamics (CFD) methods to investigate bubble-formation in the device, discussed in more detail in Section 3.3. The results from the CFD analysis found that an overall air volume fraction for the COC–PDMS original design constituted about 15%. It was observed that the flow pattern was uniform, and there were no significant perturbations during the flooding process. However, the results revealed air entrapment due to the flattened design of the chamber top cover not favoring the fully buoyant air displacement caused by the much heavier water filling up the chamber. Some recommendations to improve the design are later presented.

Generally, for liver-on-a-chip devices, due to relatively low oxygen-carrying capacity of culture medium, high perfusion rates of 100 μL·min^−^^1^ to 300 μL·min^−^^1^ are required to support 3–4 × 10^6^ HepaRG cell line [1], which, as well as Huh7 used in this work, is a hepatic cell line. Considering the count of cells loaded into the HCP chip was about 5 × 10^4^ cells, chosen flow rates (3–5 μL·min^−1^) for perfusion studies should not have limited the oxygen transport to cells from the media supply. However, in the current design, the top layer of our chip is made of PDMS, which can also serve as an additional source of oxygen penetration in the cells. Layer thicknesses of 1 and 3 mm of PDMS were tested to fabricate the HCP device to verify their suitability for this purpose.

Although silicon tubing used in flow experiments was gas permeable, 3 mm PDMS thickness was detrimental for cell growth immersed in fluid either in motion or at rest. In both cases, almost no cell attachment was observed, as shown in live/dead staining images in Figure 2d, where all non-adherent or dead cells were washed out with the eluent. In contrast, a 1 mm thickness of the PDMS layer resulted in optimal cell growth in static conditions after 24 h of cell incubation. Although some oxygen-transfer modeling would be beneficial, we speculate that the hybrid design containing the top layer of 1 mm thick PDMS has a positive impact on oxygen transport to cells from an outer environment of the chip. Additionally, the flexible nature of PDMS allows keeping the interconnect ports and connectors well aligned and attached to the surface during long-duration flow experiments. Using COC as the bottom layer versus the PC is also advantageous from a gas permeability standpoint since previous studies showed that COC was more optically transparent [3] and gas permeable than the PC was [7].

However, at the same time, despite excellent properties such as gas-permeability, ease of fabrication, and optical transparency, it is well known that PDMS has limitations, such as the absorbance of small hydrophobic molecules. These molecules are often added to the cell culture medium and the drug compounds that are regularly tested in liver-on-a-chip devices [4]. Furthermore, CFD simulations of the current design showed the entrapment of air in the top section of the chamber after the flooding of the microfluidics device containing the cell culture medium. Thus, due to the air–liquid interface aligned along the top part of the cell culture compartment, we speculate that the PDMS exposure to the cell media may be slightly minimized.

From the functionality of the HCP point of view, during fast flow rates, bubble formation in the microchannels was observed. At low flow rates, such bubble formation was not present; however, during cell culture loading or washing steps of the HCP microchannels using micropipettes, bubbles caused a significant problem. Therefore, further detailed CFD simulation modeling studies were carried out.

### 3.3. Computational Fluid Dynamics Simulations

The flooding characteristics of the microfluidic device can be pre-assessed by using a computational fluid dynamics (CFD) model. For that purpose, a computational model of the microfluidic device is analyzed using the ANSYS-CFX CFD platform. This numerical tool is based on the finite volume method to discretize and solve the Navier-Stokes equations in the Eulerian framework for the interaction of flooding fluid (water) and the air present in the microchamber. The free-surface flow is solved by implementing a compressive differencing scheme on the convection of volume fractions to keep the free surface sharp. The computation of the fluids’ volume fractions adds up to one in each control volume [34,35]. The CFD platform uses a pressure–velocity couple algorithm to solve the governing equations with greater demand in memory than a traditional segregated approach but faster convergence in parallel processing. 

Air bubbles negatively affect the performance of the cell observation process in flooded microfluidic devices [36]. Therefore, this part aimed to study the filling process of the hybrid cyclic olefin copolymer–polydimethylsiloxane (HCP) chip using CFD techniques to figure out the conditions that may diminish or eliminate the air bubbles entrapment. Hence, the main challenge of the design and optimization process of the microfluidic device object of this investigation lies in removing air bubbles that remain after the filling process is concluded.

#### 3.3.1. Governing Equations and Physical Models

Mass conservation equations for water-air and the volume-fraction weighted Navier–Stokes equations complete the governing equations to be solved under corresponding boundary conditions. Every phase was assumed as a continuum through which interpenetration may occur; both phases were considered to exist in every space position, constrained by gravity, inertial forces, and friction. The contact angle between the internal surface of the biosensor, the buffer liquid, and the air was experimentally confirmed to be approximately 90°. Thus, the capillary effect at walls was ignored during the assessment. A final equation was incorporated to ensure that a sum of volume fractions of all phases was equal to 1 in each control volume.

Governing equations are presented in the following set of equations [35,36]:

Mass Conservation:

(1) $$\nabla \cdot \left(r_{\alpha }\rho_{\alpha }U_{\alpha }\right)=0$$

Linear Momentum:

(2) $$\frac{\partial }{\partial t}\left(\rho U\right)+\nabla \cdot \left(\left(\rho U⊗U-\mu \left(\nabla U+{\left(\nabla U\right)}^{T}\right)\right)\right)=\left(B-\nabla p\right)$$

Thus,

(3) $$U_{\alpha }=U_{\beta }=U$$

(4) $$p_{\alpha }=p_{\beta }=p$$

(5) $$\rho =\sum_{2}^{\alpha =1}r_{\alpha }\rho_{\alpha }$$

(6) $$\mu =\sum_{N_{P}}^{\alpha =1}r_{\alpha }\mu_{\alpha }$$

and, for the volume fractions,

(7) $$\sum_{N_{P}}^{\alpha =1}r_{\alpha }=1$$

The buoyant force in the *z*-axis was prescribed due to the gravity and phases density difference. Equation (1) presents *α* and *β* as the water and air phases, respectively. *U* is the velocity field, *ρ* is the fluid density, *p* is the pressure field, *μ* is the fluid viscosity, and *B* is the body force (gravity only in this case). The analysis considered constant air and water properties at ambient temperature; therefore, the density and viscosity values coincide with the ambient condition.

The finite volume method discretizes the governing equations with Euler-backward time-stepping and a second-order upwind scheme in space. The discretization of the computational domain was carried out using a hybrid tetrahedron-hexahedron mesh.

Numerical simulations were performed to model the displacement of the air in the microchannel with water injected at volumetric flow rates of 5 μL·min^−1^ and 10 μL·min^−1^. The outlet boundary condition is based on a zero velocity gradient and 0 Pa gauge pressure. Thus, transient simulations with uniform *t*-steps of 0.01 s were performed to advance in time until the outlet tank was half-filled. The t-step value was chosen using a preliminary stability assessment to provide accurate and converged results.

#### 3.3.2. Computational Domain and Boundary Conditions

**Figure 3:**
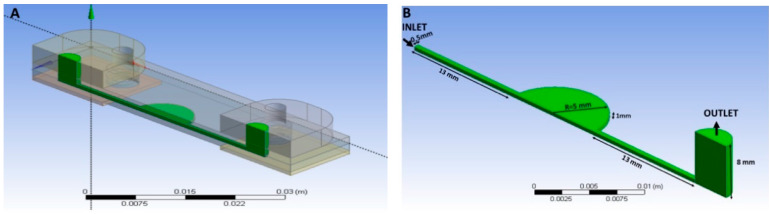
Computational domain: (**A**) 3D view of the computational domain representing half of the microfluidic domain. (**B**) Default domain.

The geometry was halved by its symmetry plane, taking advantage of such regularity in the computational domain and reducing the burden on our computational platform’s limited CPU-time and memory capacity. The computational domain was comprised of a microchannel with an outlet connector, in which water was expected to displace the air during the filling process. The inlet connector was eliminated, as shown in Figure 3. The microchannel and connector dimensions are depicted in Figure 3B.

The following dimensions: the microchannel width: 1 mm; total microchannel length: 36 mm; diameter of the central circular well (microchamber): 10 mm; and height of the connector: 8 mm, were used.

#### 3.3.3. Mesh Verification and Boundary Conditions

The computational domain was discretized with tetrahedral, hexahedral, and prismatic elements. Meshes with 83,000 (Coarse), 166,367 (Medium), and 332,734 (Fine) elements were preliminarily evaluated to assess the mesh dependency. A mesh sensitivity analysis was performed by monitoring the air volume in the microchannel including the central microchamber section. The vertical tubing connector was excluded from the calculation to estimate air entrapment within the microfluidic chamber after the flooding.

**Figure 4:**
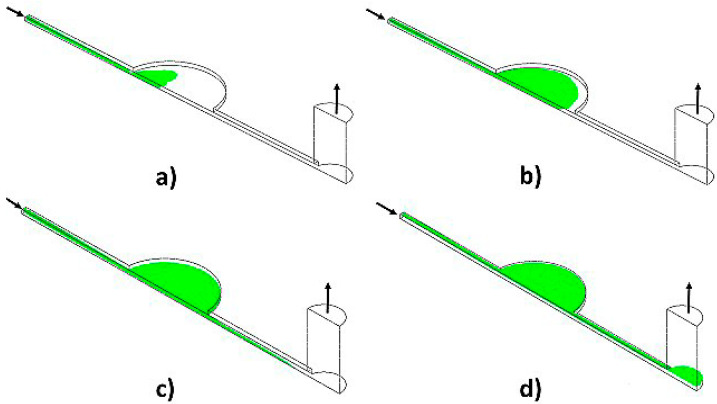
Free-surface during microchannel flooding at an inlet liquid flow rate of 5 µL/min (**a**) 20 s < τ_c_; (**b**) 35 s < τ_c_; (**c**) 65 s < τ_c_; (**d**) 330 s > τ_c_.

For the mesh sensitivity analysis, the inlet boundary condition was given by the bulk mass flow rate, which was equal to 0.83 × 10^−7^ kg·s^−1^, which corresponds to the 5 μL·min^−1^. The outlet boundary condition was set to be zero pressure and zero velocity gradient. The flooding process can be depicted in Figure 4, in which a sequential evolution of the free surface is tracked until the outflow receptacle is half-filled at the given flow rate.

The theoretical time required to fill the chip is called “critical time” (τ_c_).

Then, after water flooding the microchannel at a fixed time, a comparison between contiguous trial meshes was conducted using air fraction as a comparison parameter. After the mesh analysis, the medium-size mesh was chosen for the rest of the numerical tests. Mesh verification results and contiguous meshes deviation values are illustrated in Table 1.

The results showed 18.8% of prevalent air entrapment within the microchip control section.

The flow was relatively uniform and steady during the filling process, but a moderate free-surface waviness was observed, which might have led to the air entrapment. The large air volume fraction (0.188) at the critical time demonstrated an unsuccessful filling process. A similar pattern was observed for a flow rate of 10 μL·min^−1^, showing an overall air volume fraction of 0.245 at a critical time for that flow rate condition. It demonstrates that an increase in inlet flow rate is directly proportional to the air volume entrapped in the current design.

In an attempt to reduce the air entrapment and use the HCP design (Figure 1b) as the primary design for experimental studies, the model’s height was reduced to 0.5 mm. After rerunning the CFD simulation, the resulting air volume fraction after flooding was 0.153, demonstrating the positive effect of reducing the height of the chamber. This trend clearly shows that the level of free surface unsteadiness was the critical factor in air entrapment since the higher the height of the chamber, the more significant the amplitude of the surface waves during the flooding process. A further enhancement in the chamber configuration was introduced to help the air release during the process. The numerical model assumed a slight inclination upward in the chamber towards the outlet to favor the natural buoyant migration of the air during the flooding. The simulation results under such conditions showed a significant decrease in the air-volume fraction (0.0032 for 10° and 0.0007 for 5°) after the critical time was reached. Experimental validation conducted on the inclined surface has shown similar results; air bubbles were minimized by inclining the device, making air trapping much more difficult to happen throughout the flooding process.

Experimentally, the CFD simulation results improved the cell loading and washing procedures of the HCP while using a pipette preventing formation of bubbles in the central well. Physical slight tilting of the device during loading procedures for small angles with the positive slope allowed to avoid any bubble formation confirming the modeling results. The simulation results benefited from the successful experimental collagen-coating and cell loading procedures. A continuous flow through the device did not show any bubble-entrapment during perfusion studies and wash-out procedures using a syringe pump at a low flow rate. It allowed us to run perfusion with the device in a horizontal orientation without inclination.

### 3.4. Perfusion of Cell Culture in the HCP Platform

The functional and metabolically active hepatocytes cultured under perfusion are critical for drug testing applications of liver-on-a-chip (LOC) platforms. The initial source of hepatocytes loaded into a microfluidic chip for LOC applications may vary. Freshly isolated liver cells or cryopreserved primary hepatocytes (PH) are considered to be the gold standard in drug toxicity and are widely preferred for drug metabolism studies [37]. However, they may provide inaccurate and not reproducible results due to problems with their extraction, donor-to-donor variability, and de-differentiation [38]. The immortalized cell lines are often preferred in LOC platforms since the reproducibility of experimental results is crucial [24]. Commercially available cell lines, such as Huh7, enable a more stable and reproducible cell source of human cells for LOC development.

Several liver-on-a-chip platforms reported recently were based on similar hepatic cell lines such as HepaRG, HepG2/C3A [39,40]. The most widely used cell line for conventional hepatoxicity testing is HepG2, while Huh7 cells used in the study have shown lower metabolic capacity [41]. Since in this work we focus on cell functionality and growth in the developed hybrid COC–PDMS device and not on drug metabolism studies, the Huh7 cultured COC–PDMS platform holds great potential for developing a robust and reproducible liver-on-a-chip that can be applied to other types of cells for drug metabolism studies.

Continuous perfusion was carried out under a 3 μL·min^−1^ flow rate to evaluate the ability of the HCP device to culture cells over time. The functionality of liver cells in the device was evaluated by assessing the levels of important biomarkers, such as albumin and urea, that indicate the metabolic activity and functionality of cells [42,43,44]. Levels of the biomarkers were assessed during different time points up to 24 h of culturing in the device under constant perfusion.

**Figure 5:**
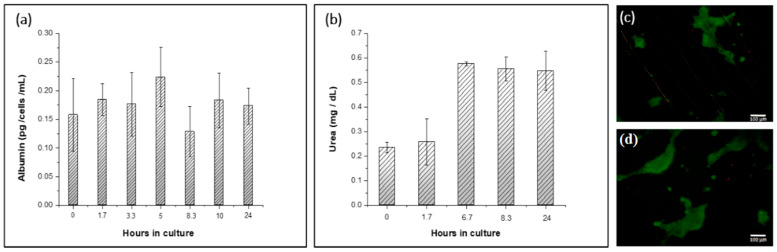
(**a**) Albumin secretion rates generated by a hybrid cyclic olefin copolymer–polydimethylsiloxane (HCP) microfluidic platform applied for Huh7 hepatoma cell line culture under perfusion; (**b**) Secretion rate of urea collected in eluent fractions from the HCP over 24 h at 37 °C; (**c**,**d**) Live-dead assay imaging results from HCP device with 1 mm thickness of PDMS layer. Green (Calcein-AM)—live cells, red (Propidium Iodide)—dead cells (no autofluorescence from the substrate).

The COC–PDMS cultured cells demonstrated relatively stable albumin secretion up to 24 h, as shown in Figure 5a. A slight increase in albumin production can be noticed at 6 h and onwards of perfusion culturing. It is reported in the literature that the function and activity of cells can fluctuate over time [39,45]. Khedr et al., for example, reports a similar pattern for the Huh7 bioreactor and 2D cell monolayers from Huh7 cells, for which albumin concentrations showed an increase at the 6 h time mark [46]. We, therefore, speculate that slight fluctuation in the levels is due to the variations in cell growth and interactions over time and cell adaptation to the flow conditions, which may have affected functionality, and this variation was in line with the reported literature.

Furthermore, albumin levels were comparable to the rates secreted by Huh7 cells reported in the literature. The albumin production from the HCP device used for culturing Huh7 cells in a continuous mode was (5.57 ± 0.24) ng·mL^−1^ in the collected volume fractions. For control samples cultured in static conditions, albumin production was determined to be (189.16 ± 36.44) ng·mL^−1^ cultured within the 24 h period. In comparison, Khedr et al. reported (11.02 ± 2.18) ng·mL^−1^ albumin concentrations after 6 h of culture in the developed acoustofluidic bioreactor based on Huh7 cells and (5.62 ± 0.85) ng·mL^−1^ with cells seeded as 2D monolayers at the 3 h mark, which then almost doubled at the 6 h time mark [46].

The biomarker concentrations were calculated in terms of pg·cells^−1^·mL^−1^·h^−1^ to compare the albumin concentrations to more studies reported in the literature. The albumin secreted by the cells in the device was (0.11 ± 0.02) pg·cells^−1^·mL^−1^·h^−1^. Here, the amount of albumin was within the range reported in the literature. For example, HepG2 cells/spheroids secrete (0.6–80) pg·cells^−1^·day^−1^ [47,48]. The bioreactor containing HepG2 spheroids was reported to produce (20 ± 3) pg·cell^−1^·day^−1^ in the first 24 h [39]. Considering HepG2 is a more metabolically active cell line than Huh7 [41], albumin levels produced in our HCP device suggested good functionality of the perfusion culture in the platform.

Additionally, urea synthesis was found within the norms or even higher than those reported in the literature for Huh7 cells. Urea is the major nitrogenous end-product of protein metabolism in mammals [49], which is also used as a parameter to determine hepatocyte viability [50]. Thus, analysis of this parameter from flow-through fractions collected during perfusion from the HCP device was another indicator of the Huh7 functionality under continuous flow. Levels of secreted urea by the cultured cells in the device showed slight fluctuation at the beginning of the culture and stabilized over time (Figure 5b). The average value of urea in collected samples was (0.43 ± 0.04) mg·dL^−1^ during the perfusion experiments. These values in converted units were comparable to those reported in the literature too. For example, Huh7 microtissues assessed after 1 day of culture show (70–80) μg·mL^−1^·24 h^−1^ levels of urea [51]. Here, urea levels almost doubled after the first 4 h of perfusion culture in the HCP. This pattern is also reported in the literature [46], where Huh7 cells cultured in the bioreactor and 2D culture forms had a time-dependent increase in urea secretion following 6 h in culture. Passing the first 6 h perfusion time mark, urea secretion concentrations in the collected fractions stabilized, suggesting that the cell functionality and viability in the hybrid device remained stable and within the norms.

The hybrid COC–PDMS device microchannel was perfused with a live/dead cell staining kit solution for microscopy imaging to confirm the cell viability. Figure 5c,d shows the images of cells in the microchannel where live (green) cells dominate, suggesting that the viability of cells after 24 h of continuous flow was stable and perfusion did not inhibit and possibly enhanced cell growth. Cells formed small aggregates within the microchannel during the staining procedure, but supernatant analysis on the presence of cells showed no detached or washed out cells. A significantly higher number of cells was observed after perfusion experiments during microscopy imaging. Biomarker investigations and live/dead cell imaging assays suggested that perfusion Huh7 cell culture remained intact and metabolically active throughout the continuous flow experiments.

Overall, optical and fluorescence microscopy showed confluent and intact cell culture before and after a continuous flow was carried out through the LOC (Figure 2a and Figure 5c). The cells remained attached to the COC chamber surface. A short evaluation study was carried out to assess the functionality of the Huh7 culture in the LOC. Equal volume fractions of the flow-through media were collected. Albumin and urea production per cultured number of cells, time, and volume of the measured sample was comparable and found to be in the range of values of albumin secreted in traditional 2D static cell cultures (Figure 5a,b). Although the functionality of the LOC cell culture in the perfused system needs to be investigated further, this work provides an initial proof-of-concept demonstration of the utility of the developed LOC device platform, which will be applied for drug metabolism assessment in future studies.

## 4. Conclusions

Liver-on-a-chip (LOC) microfluidic platforms recapitulating certain organ characteristics can help accelerate the drug development field and reduce animal models used in drug testing. However, to create robust LOC platforms, it is essential to use materials suitable for the growth of functional cell culture under perfusion. In contrast to single-substrate devices, the number of hybrid platforms applied for cell culture applications has grown in recent years [15]. PDMS-based hybrid devices allow leveraging oxygen permeability of PDMS for cell culture while avoiding its limitations, such as nonspecific adsorption of small molecules, leaching of molecules, swelling or shrinking at the contact with many organic solvents [15]. In combination with PDMS, employing cyclic olefin copolymer thermoplastic, which has excellent properties suitable for on-chip cell culture applications, can open doors towards creating a robust LOC system. We described the development of a hybrid cyclic olefin copolymer and polydimethylsiloxane (HCP) microfluidic platform for growing a functional perfusion cell culture of the Huh7 hepatoma cell line that takes advantage of both materials while minimizing their shortcomings. Design and material considerations based on polymers widely used for microphysiological platforms were evaluated. Based on the experimental results and microscopy imaging observations, the developed HCP device chip was found to be optimal among the other evaluated platforms for cell growth and the reusability of the device. Computational fluid dynamics simulations were carried out to analyze the bubble formation in the device and the flow distribution. The fabrication method was simple, resulting in a stable fluidic system applicable for perfusion cell culture for 24 h. The concentrations of biomarkers in cell culture were analyzed to evaluate the Huh7 perfusion cell culture in the developed HCP. The secretion rates of albumin and urea, and cell viability after perfusion showed that the cell culture in the HCP remained functional and metabolically active for the duration of the study. The response of the perfusion culture in the HCP was similar to in vitro models, showing its potential application for liver-on-a-chip development. The HCP platform can be applied to any other cells widely used in LOC platforms (HepG2, HepRG, or primary human hepatocytes). The optimization of the functionality of cell culture in the HCP device and its application in drug metabolism studies can pave the path towards developing a robust and accurate LOC system.

## Figures and Tables

**Table 1:** Mesh dependency analysis.

Mesh-Elements (Feature)	Air Fraction	Deviation, %
41,000 (XX-Coarse)	0.156	
83,000 (Coarse)	0.187	16.6
166,367 (Medium)	0.188	0.55
332,734 (Fine)	0.188	0.16

## Data Availability

Data are contained within the article or Supplementary Materials.

## Supplementary Materials

The following are available online at https://www.mdpi.com/article/10.3390/polym13193215/s1. Figure S1: Brightfield images of cells on PC, PS. Figure S2: Images of cell growth on PDMS. Figure S3: Images of cell growth on COC. Figure S4: Cross-section view.
