# Supplementary material for: Development of a Hybrid Polymer-Based Microfluidic Platform for Culturing Hepatocytes towards Liver-on-a-Chip Applications
Source: Polymers (Basel). 2021 Sep 23;13(19):3215. doi: 10.3390/polym13193215 (PMC8513053; doi:10.3390/polym13193215)
Supplement: Supplementary file 1 [file polymers-13-03215-s001.zip › supplementary materials.pdf]

polymers

MDPI

Supplementary Materials

1. Collagen I Surface Coverage Calculations

Concentration of the stock solution was 0.1% (1 mg/mL) as stated by the manufacturer (Sigma-Aldrich)

Following dilution in sterile diH_2O, the final solution was 0.1 mg/mL or 0.1 µg/µL. The calculated volume of the chip without connectors was 64.81 µL. To calculate the surface coverage, the volume of the chamber was multiplied by the collagen concentration and further on divided by the chamber area (1.2962 cm^2).

$$\frac{64.81\ \mu L * 0.1\ \mu g/\mu L}{1.2962\ cm^2} = 5.0\ \mu g/cm^2$$

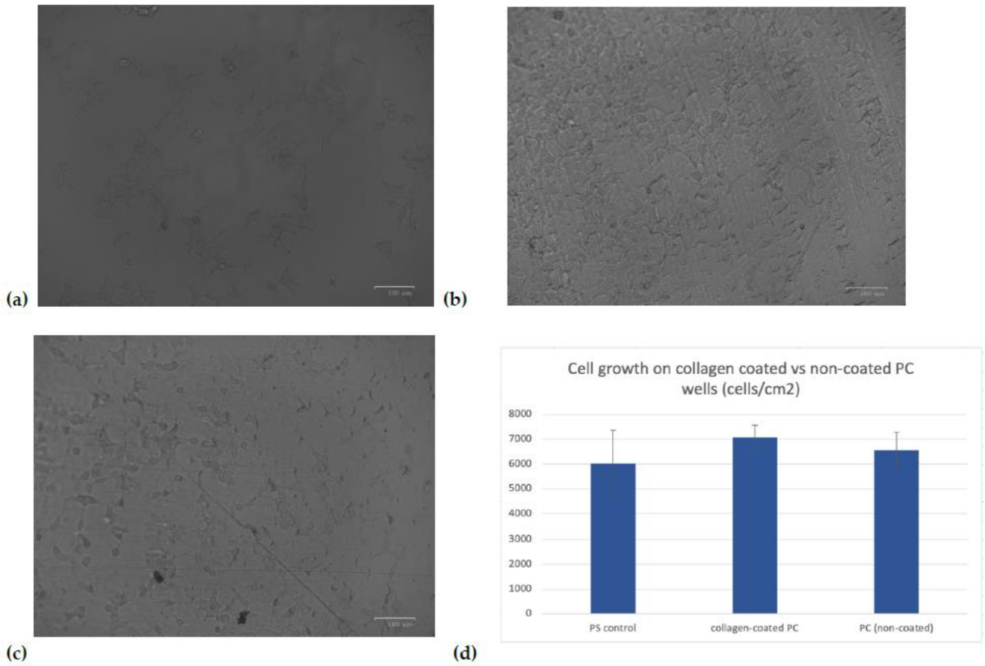

Figure S1. Brightfield images of cells on PC, PS. The cells on collagen-coated PC (**b**) demonstrated 117.8% growth of polystyrene control (**a**) and 108.1% of non-coated PC well (**c**).

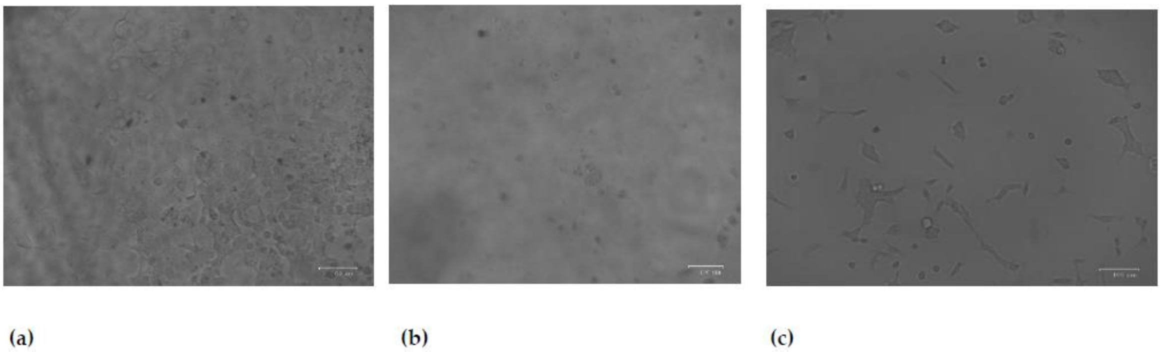

Figure S2. Images of cell growth on PDMS. (**a**,**b**) Cell growth on polydimethylsiloxane (PDMS) layer. (**c**) Cell growth on control bare polystyrene dish.

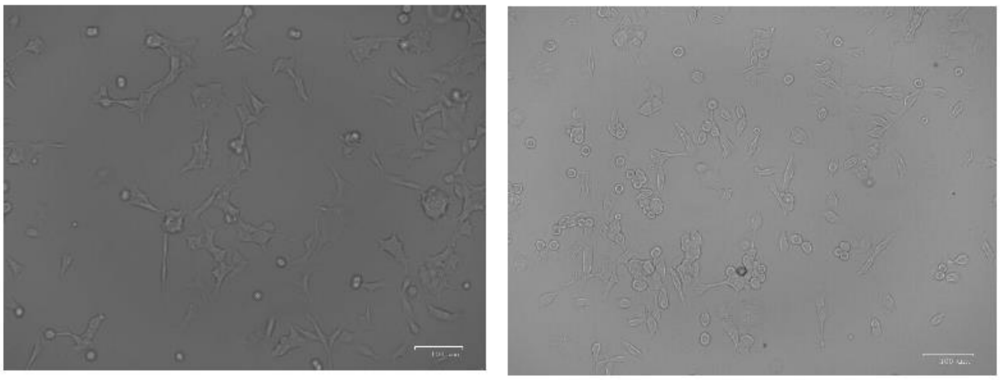

(a) **(b)**

Figure S3. Images of cell growth on COC. (**a**) Cell growth on collagen-coated COC layer. (**b**) Cell growth on collagen-coated polystyrene dish.

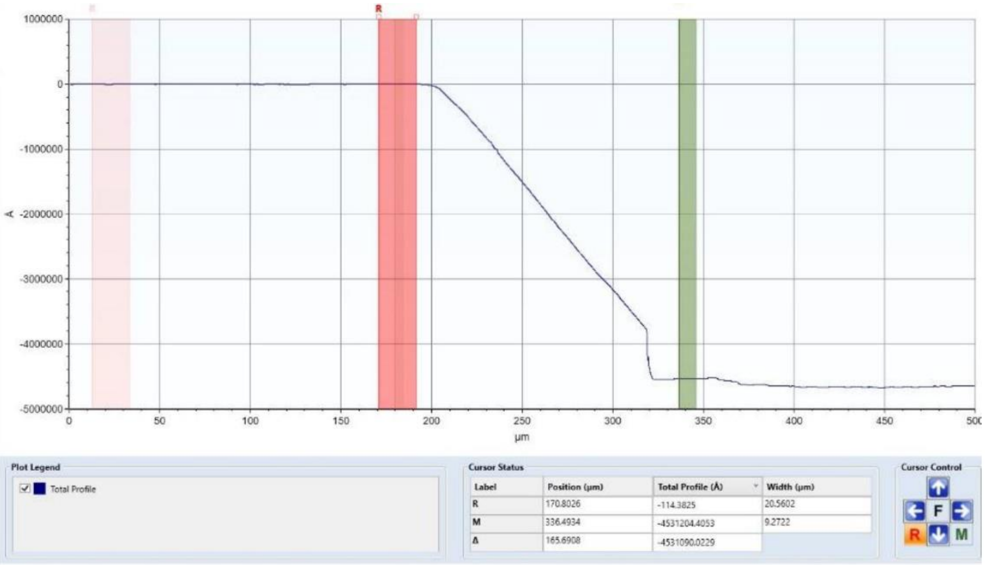

Figure S4. Cross-section view. The profile of the microchannels surface measured by a contact profilometer. The actual depth of the microchannel was 450 µm.
